# Developing and Implementing a Pediatric Emergency Care Curriculum for Providers at District Level Hospitals in Sub-Saharan Africa: A Case Study in Kenya

**DOI:** 10.3389/fpubh.2017.00322

**Published:** 2017-12-11

**Authors:** Colleen Diane Fant, Kevin R. Schwartz, Hiren Patel, Karla Fredricks, Brett D. Nelson, Kennedy Ouma, Thomas F. Burke

**Affiliations:** ^1^Department of Emergency Medicine, Ann and Robert H. Lurie Children’s Hospital of Chicago, Chicago, IL, United States; ^2^Department of Pediatrics, Massachusetts General Hospital for Children, Boston, MA, United States; ^3^Division of Global Health and Human Rights, Department of Emergency Medicine, Massachusetts General Hospital, Boston, MA, United States; ^4^Department of Emergency Medicine, Massachusetts General Hospital, Boston, MA, United States; ^5^Department of Pediatrics, North Shore Medical Center, Salem, MA, United States; ^6^Maseno University, Maseno, Kenya

**Keywords:** pediatric emergency medicine, pediatric emergency care, low-income, rural, sub-Saharan Africa, Kenya, curriculum

## Abstract

**Introduction:**

Emergency medicine is a relatively new field in sub-Saharan Africa and dedicated training in pediatric emergency care is limited. While guidelines from the African Federation of Emergency Medicine (AFEM) regarding emergency training exist, a core curriculum in pediatric emergency care has not yet been established for providers at the district hospital level.

**Methods:**

The objective of the project was to develop a curriculum for providers with limited training in pediatric emergencies, and contain didactic and simulation components with emphasis on treatment and resuscitation using available resources. A core curriculum for pediatric emergency care was developed using a validated model of medical education curriculum development and through review of existing guidelines and literature. Based on literature review, as well as a review of existent guidelines in pediatric and emergency care, 10 core topics were chosen and agreed upon by experts in the field, including pediatric and emergency care providers in Kenya and the United States. These topics were confirmed to be consistent with the principles of emergency care endorsed by AFEM as well as complimentary to existing Kenyan medical school syllabi. A curriculum based on these 10 core topics was created and subsequently piloted with a group of medical residents and clinical officers at a community hospital in western Kenya.

**Results:**

The 10 core pediatric topics prioritized were airway management, respiratory distress, thoracic and abdominal trauma, head trauma and cervical spine management, sepsis and shock, endocrine emergencies, altered mental status/toxicology, orthopedic emergencies, burn and wound management, and pediatric advanced life support. The topics were incorporated into a curriculum comprised of ten 1.5-h combined didactic plus low-fidelity simulation modules. Feedback from trainers and participating providers gave high ratings to the ease of information delivery, relevance, and appropriateness of the curriculum.

**Conclusion:**

We present here a core curriculum in pediatric emergency care for district hospital level providers in Kenya which can be used as a framework for further development and implementation of training programs throughout sub-Saharan Africa.

## Introduction

Emergency medicine (EM) is a relatively new field in sub-Saharan Africa. Despite the region’s high burden of disease from acute illness, the EM model of care is still in its nascence, as is specific training in this field (1–4). The first EM postgraduate training for physicians was developed in South Africa at the University of Cape Town in 2004 (1). Since then, similar programs have been implemented in Botswana, Rwanda, Ethiopia, Tanzania, and Ghana (2, 5, 6). However, to date, dedicated postgraduate training in the field of pediatric emergency medicine (PEM) is not available in sub-Saharan Africa (7). While training in pediatric emergencies is included as part of pediatric training or EM training, a dedicated curriculum focused on the core knowledge and skills requisite to the practice of emergency pediatric care in the district hospital setting does not exist.

Approximately 25% of pediatric patients presenting to rural and urban health facilities in Africa have a high acuity disease (8). Several studies demonstrate that a majority of causes of morbidity and mortality could likely be reduced by more effective emergency care (5, 8, 9). As such, improvements to pediatric emergency care have the potential to impact in-hospital mortality significantly. Current efforts to improve the care for acutely ill adults and children have focused on prehospital care systems, the development of emergency triage and stabilization facilities, community health worker programs, increased medical capacity with nurses and mid-level providers, and physician training (2, 9). Training for mid-level and non-EM physician providers in the specifics of pediatric emergency care is one aspect of these efforts which has not been well standardized.

While EM postgraduate training opportunities are growing in Africa, the majority of emergency care is still provided by physicians and mid-level practitioners with no formal EM training and little pediatric specific training (5, 9, 10). In Kenya, there are very few physicians with formal training in EM. There is currently no postgraduate training in EM available in Kenya for physicians, though several are in development. Most emergency care in Kenya is provided by clinical officers who do not have specialized qualification or certificates in EM (11). Kenya is a lower middle income country with an under-five mortality rate of 73 per 1,000 children (2012) an infant mortality rate of 49 per 1,000 (2012), and a prevalence of moderate or severely underweight at 16.1% of children under 5 years of age (2012) (12). Most inpatient hospital mortality occurs within 24–48 h of admission, and 80% of child mortality has been attributed to few and treatable causes including infection, malnutrition, and newborn etiologies (13). The disproportionate burden of acute childhood illness in Kenya, coupled with a lack of providers with specific training in pediatric emergency care at the district hospital level, highlights the need for an established standardized curriculum in this area to train physician and non-physician providers working in the field. Several Kenyan universities have begun to recognize the gap in EM training and develop programs. At Maseno University in Kenya a new residency program in Family and Emergency Medicine was established in 2014 as a partnership between Maseno and the Massachusetts General Hospital (MGH) Center for Global Health and Human Rights. The launch of this new partnership provided a unique opportunity to focus on one critical aspect of EM training; pediatric emergency care. We present here the development of a core curriculum for training in pediatric emergency care at a district level hospital.

## Methods

### Framework

To guide the development of this curriculum, the model of curriculum development for medical education was used (Appendix A) (14).

### Learning Environment

The targeted learners for this curriculum were all current clinicians at Sagam Community Hospital (SCH) affiliated with Maseno University School of Medicine. At the time of curriculum development there were three Kenyan residents in the FEM program and four clinical officers working in the emergency department at SCH. Both residents and Clinical Officers had a minimum of 2 years of clinical experience as general health care providers. All residents hold an MBBS from a Kenyan medical school and had previously completed an internship. All clinical officers held a Diploma in Clinical Medicine from a Kenyan Medical Training College.

The setting for the implementation of the curriculum was SCH in western Kenya. The hospital is one of the primary teaching sites for a 4-year FEM residency program. At the hospital, a dedicated emergency department with pediatric resuscitation capabilities was established in April 2015. A shipping container on the hospital grounds serves as the classroom for educational activities with a small area for practicing of clinical skills and scenarios with a pediatric and newborn size mannequin. A full medical simulation center is currently under construction at SCH.

### Educational Standards

For both the general and targeted needs assessment, in addition to work with the partnership between MGH and Maseno, a literature review was performed to identify current educational strategies in sub-Saharan Africa and internationally and identify best practices.

In order to identify standards and competencies to be addressed in the curriculum, a review was performed to identify literature relevant to pediatric emergency care. A PubMed search was conducted using the search terms: “international pediatric emergency medicine,” “pediatric emergency medicine education,” “global health and pediatric education,” “international emergency medicine,” and “global health and emergency medicine education.” In addition to the literature identified through PubMed, known existing guidelines for the care of acutely ill pediatric patients in high, middle and low-income countries were reviewed. These sources included: the American Board of Pediatrics core competency list for PEM (15), consensus statements from the African Federation of Emergency Medicine (AFEM) (2), the AFEM Handbook of Acute and Emergency Care (16), guidelines and training resources from the World Health Organization (17) [including the training manuals for the emergency triage, assessment, and treatment (ETAT) (18) and ETAT+] (13), the Kenyan Basic Pediatric Protocols (19), the International Federation for Emergency Medicine (IFEM)’s curriculum for medical students (20), existing curricula from an EM program in Liberia (21, 22), and the 2012 International Standards of Care for Children in Emergency Departments published by IFEM (23).

Based on the initial literature and guideline review, a tentative list of 15 core topics was developed and reviewed by a panel of multiple experts and potential stakeholders. Participants included specialists in pediatrics and EM as well as US-trained pediatric and PEM providers with significant experience working at multiple training sites in resource-limited settings. These topics were then electronically presented to members of AFEM as well as Kenyan physicians specialized in EM, Pediatrics, or Family Medicine. Topics were compared to and designed to be complimentary to the existing syllabus for the Maseno University School of Medicine program. Feedback was incorporated to determine the final list of 10 topics comprising the core curriculum.

For each of the subtopics, additional literature review was performed to ensure that evidence-based practices were being taught and to complement the information used to derive the overall curriculum. To address discrepancies between the existing guidelines, resources such as ETAT/ETAT+ and AFEM that were specifically created for clinical practice in sub-Saharan Africa were given more weight than international or American practices. Additionally, resources that had the most relevance to practice limitations at SCH were prioritized in creating the didactic portions of the subtopics.

### Curriculum Goals and Objectives

The goal of the curriculum was to develop a standardized set of topics in pediatric emergency care for the training of providers at district level hospitals. Objectives included the development and implementation of topic-based modules that contained didactic lectures and lo-fidelity simulation experience in areas of pediatric emergency care that are both common and rare, with emphasis on treatment and resuscitation using available resources.

### Educational Format

The curriculum consisted of ten 1.5-h combined didactic/case simulation modules. Modules were created by US pediatric residents at MGH with oversight by EM and Pediatric faculty in the US and Kenya. The content and learning objectives of each module were adapted to include the specific treatment modalities available at a rural district level hospital in Kenya as well as resources available at larger, urban referral hospitals to prepare the learners for additional practice environments. The modules were led by different US-trained educators from MGH from the Department of Global Health and Human Rights who work at SCH in a long-term capacity.

This curriculum was then piloted with participating clinical officers and FEM residents at SCH. The 10 modules of the curriculum were taught to three Kenyan FEM residents and four clinical officers during their weekly established didactic time from June 2015 through January 2016. The curriculum was integrated into the overall longitudinal residency education rather than given as an intensive course of several days in order to utilize existing time set aside for education and minimize taking the trainees away from patient care. Modules that included simulation cases were held immediately following didactic portions, using a pediatric mannequin and relevant medical supplies borrowed from the active emergency department. Following each module, structured written and verbal feedback on content and perceptions of the lesson was collected from the participants and facilitators.

## Results

The PubMed search resulted in 75 articles. Fifteen of these were excluded based on title. The remaining 60 articles were then reviewed by abstract and title, with 23 deemed relevant by two authors (Colleen Diane Fant and Kevin R. Schwartz) and subsequently reviewed in full.

The topics constituting the core curriculum, derived from the literature review, guideline review, and expert consensus, are listed in Table 1.

**Table 1:** Core topics in pediatric emergency care.

Core topics in pediatric emergency care
Pediatric airway management
Respiratory distress in the child
Pediatric thoracic and abdominal trauma
Pediatric head trauma and cervical spine management
Pediatric sepsis and shock
Pediatric endocrine emergencies
Pediatric altered mental status and toxicology
Pediatric orthopedic emergencies
Burn and wound management
Pediatric advanced life support

Table 2 details the syllabus outline used for each of the 10 curriculum modules. Each module either integrates multiple interactive verbal cases into the didactic portion or includes 1–2 low-fidelity simulations using a pediatric or newborn mannequin following the didactic portion.

**Table 2:** Syllabus.

**Module 1: Pediatric Airway Management** ABCs Manual airway management Anatomic considerations Technique for jaw thrust and chin lift Management of choking Oxygen delivery systems Nasal cannula Non-rebreather mask CPAP/bubble CPAP Ambu bag Airway adjuncts Nasopharyngeal airway Oropharyngeal airway Intubation and indications Rapid Sequence Intubation (RSI) Medications and dosing Resource specific considerations Interactive case presentation 3-year-old female with foreign body in airway Mannequin Simulations 7-year-old female with respiratory distress due to pneumonia 5-year-old male with airway compromise secondary to seizure activity Debriefing of Simulations

**Module 2: Respiratory Distress in the Child** Identify major differences between children and adults with respiratory distress Form a systematic initial evaluation of illness severity Review of physical exam findings in children with respiratory illness Interactive case presentations: upper versus lower airway findings Review of ancillary studies Interactive case presentations: common pediatric causes of respiratory distress and management Laryngotracheobronchitis Bacterial tracheitis Epiglottitis Complications of pharyngitis: peritonsillar abscess and retropharyngeal abscess Acute management of choking and foreign body aspiration Pneumonia and complicated pneumonia Bronchiolitis Asthma Anaphylaxis Review of commonly used medications and dosing Mannequin simulations: Laryngotracheobronchitis Asthma exacerbation Debriefing of simulations

**Module 3: Pediatric Trauma: General Approach and Thoracic and Abdominal Trauma** Local epidemiology Road traffic accidents Systematic approach: advanced traumatic life support Primary survey: airway, breathing, and circulation Shock Disability and exposure Secondary survey Overview of types of abdominal trauma Management of blunt abdominal trauma Liver and splenic lacerations and management Hollow viscera injury Use of imaging Overview of types of thoracic trauma Lung contusions Rib fractures Pneumothoraces—technique for needle decompression Hemothorax Esophageal injury Aortic injury Hemopericardium—technique for pericardiocentesis Tetanus prophylaxis Interactive case presentations 7-year-old male victim of road traffic accident with tension pneumothorax 10-year-old female, unresponsive, victim of road traffic accident 4-year-old male with blunt abdominal trauma Debriefing

**Module 4: Pediatric Trauma Management: Head Trauma and Cervical Spine** Review of the principles of trauma assessment ABCDE Initial survey Cervical spine injury Anatomic considerations Stabilization techniques Clearance Secondary survey Head trauma Physical exam findings Concussion, fracture, bleeds, and diffuse axonal injury Management of elevated intracranial pressure Interactive case presentations: 5-year-old male with head trauma 4-year-old male with low risk head trauma 12-year-old male with neck pain following road traffic accident 9-month-old male with altered mental status secondary to non-accidental trauma Discussion/questions

**Module 5: Pediatric Sepsis and Shock** Define sepsis and shock How to recognize shock quickly Distinguish between different types of shock Basic management strategies of shock Fluid resuscitation with and without severe malnutrition Use of vasopressor support Review of normal pediatric vital signs Review of potential lab abnormalities seen in shock Interactive case presentations: 6-month-old male with septic shock 7-month-old female with cardiogenic shock secondary to supraventricular tachycardia 9-year-old female with obstructive shock secondary to tension pneumothorax 6-year-old male with hypovolemic shock secondary to trauma Discussion/questions

**Module 6: Endocrine Emergencies: Diabetic Ketoacidosis (DKA) and Adrenal Crisis** Epidemiology of DKA in sub-Saharan Africa Pathophysiology of DKA Recognition and diagnosis of DKA Signs and symptoms Expected laboratory abnormalities Complications of DKA Emergency department management of DKA Adrenal insufficiency Pathophysiology and etiology of adrenal insufficiency Recognition and diagnosis of adrenal crisis Management of adrenal crisis Presentation of congenital adrenal hyperplasia Interactive case simulation: 6-year-old male with DKA Discussion/questions

**Module 7: Altered Mental Status and Pediatric Toxicology** A systematic approach to the pediatric patient with altered mental status Review of the differential diagnosis for altered mental status in the child Review of basic management of seizures in a pediatric patient Review of the common pediatric toxidromes and their management Mannequin simulation Simple febrile seizure Mannequin simulation Hypoglycemic seizure Debriefing of simulation cases

**Module 8: Pediatric Orthopedic Emergencies** Review of the differences between adult and pediatric musculoskeletal injuries Review of bone development, ossification centers, Salter-Harris fractures Review of basic approach to a fracture or musculoskeletal injury Interactive case presentations of common pediatric orthopedic injuries with x-rays on projector screen: Long bone fracture Compartment syndrome Fall onto outstretched hand Greenstick fracture Buckle fracture Radial head subluxation, “Nursemaid’s Elbow” Supracondylar fracture Clavicle fracture Toddler’s fracture Spiral femur fracture Fractures that require immediate orthopedic referral The child who refuses to bear weight on lower extremity Discussion/questions

**Module 9: Pediatric Burn and Wound Management** Background and epidemiology of pediatric burns Pathophysiology of burns Classification and management of burns Specific medications for burn management Additional considerations in burns: ABCDs Useful labs When to admit, discharge, or transfer patients Specific burn types Infection management Escharotomy Utilization of Parkland Formula for fluid management Principles of wound management Interactive case presentations with photographs of burns on projector First- through fourth-degree burns Discussion/questions

**Module 10: Pediatric Advanced Life Support (PALS)** Review of ABCD and overall approach Review of normal pediatric vital sign ranges by age Basic life support skills: giving breaths and doing compressions Review of important resuscitation medications and dosing Pediatric Cardiac Arrest Algorithm Reversible Causes of Cardiac Arrest (H’s and T’s) Ventricular fibrillation/Ventricular tachycardia/Asystole/Pulseless electrical activity Pediatric Bradycardia algorithm Common EKG findings Pediatric tachycardia algorithm Post-resuscitation care Mannequin simulations 3-year-old female found down (pulseless arrest) 5-year-old male with bradycardia Management of supraventricular tachycardia Debriefing of simulations

### Assessment and Feedback

The modules and overall curriculum were reviewed positively by participants. On a 5-point Likert scale, participants were asked to rank their agreement (maximum score 5) or disagreement (minimum score 1) with a series of questions regarding the curriculum. In response to the survey, the participants agreed with the following three statements: “The topic was relevant to my clinical practice” (mean Likert score of 4.5 out of 5), “The lecture was at the appropriate academic level for my learning” (mean Likert score of 4.4 out of 5), and “The lecture materials were helpful” (mean Likert score of 4.4 out of 5). Comments in unstructured written feedback were particularly positive about the case simulation component of the curriculum, and suggestions for curriculum improvement included having less lecture time and more case-based simulation scenarios. Future evaluation of the curriculum, including posttraining knowledge assessment, participant skill observations, or patient outcomes have not been done to evaluate the effectiveness of the curriculum.

## Discussion

Postgraduate EM training is growing in sub-Saharan Africa. However, the majority of care for pediatric emergencies is still provided by practitioners without specific EM training. Given the high proportion of pediatric patients presenting to district level hospitals in limited-resource settings, it is critical to develop specific pediatric emergency care training for clinicians taking care of these patients (8). Here, we describe a standard curriculum for clinicians working with acutely ill pediatric patients in limited-resource areas and demonstrate the feasibility of implementing a curriculum with clinical officers and residents at a community hospital in western Kenya.

While there are several published resources for the training of clinicians in the emergency care of children, a set of pediatric emergency care core competencies for physicians and mid-level providers has not previously been published and a modular and replicable core curriculum does not exist. The core curriculum described here is drawn from a wide range of national and international published guidelines, scientific articles, and expert consensus and was developed specifically for practicing providers in resource-limited district hospital level settings. Future directions could include transitioning this program evaluation toward a competency-specific model using the reviewed topics. Although the accompanying curriculum was tailored to the resources available at a district hospital level, it also includes best practices for settings with additional resources, with an eye toward educating providers for the future as available medical resources expand. This curriculum addresses a training gap but does not address the infrastructure gap that often exists in limited resource settings and future efforts may also include evaluation and recommendations of minimal physical resources required to treat pediatric emergencies.

The entirety of pediatric emergency care clearly cannot be reduced to a list of 10 topics and, indeed, US physician training for board certification in PEM requires a 2- to 3-year fellowship program at an accredited institution. Yet, the purpose of the core topics here established is to define the minimal, critical topics in which providers of emergency care should have training in order to provide an acceptable standard of emergency care to children. The accompanying curriculum is appropriate for both physician and mid-level providers and was well received by both groups of learners. This curriculum is not comprehensive with regards to pediatric emergency care but provides a foundational level of training in the evaluation of initial management of acutely ill children.

Our curriculum incorporates significant use of medical simulation and case-based interactive discussion. Verbal and written feedback from participants cited this as one of the most useful and well-liked aspects of the curriculum. The use of hands-on simulation and interactive case examples to optimize participants learning and attention is in keeping with adult learning best practices and is supported by existent medical education literature. It represents a key component of this curriculum in engaging and activating participants. Further studies could use the simulation aspect of the curriculum to have participants demonstrate competency in key skills and medical decision making.

The initial pilot implementation of this curriculum had several limitations. First, the literature review and curriculum are in English, which limits their applicability in non-English speaking medical settings. All of the resources used were in English, and many were adapted from US-based programs. Additionally, the number of participants was relatively small, limited to three residents and four clinical officers. As such, the results cannot be generalized to other types of providers or to a larger number of providers. Further, the curriculum was only implemented at a single site and, thus, its applicability to other hospital settings has not been established. Lastly, assessment of the curriculum was limited to learners’ attitudes regarding the curriculum and did not evaluate whether knowledge was gained through participation in the curriculum, whether such knowledge affected practice, competence demonstrated, or whether patient outcomes were improved by participation in this curriculum, which is its ultimate goal.

Future efforts could expose a wider group of participants to the curriculum as well as assess pre- and postcurriculum knowledge and/or changes in practice which may result from participation in this curriculum as well as to identify learning objects for different level providers. Evaluation could include pre- and postimplementation performance in simulation sessions or objective structured clinical examinations (OSCEs). Assessment of the clinicians’ care of patients before and after training could be used to identify changes in practice resulting from participating in this curriculum.

## Conclusion

Here, we present core topics in pediatric emergency care developed from existent guidelines, literature review, and expert consensus, along with an accompanying curriculum to train physicians and mid-level providers in them. Our initial pilot of this curriculum was well received by learners and facilitators. This curriculum may be adapted and replicated at similar training sites in limited resource settings in sub-Saharan Africa. As emergency care continues to expand in sub-Saharan Africa, these competencies represent one means to ensure that hospitals providing emergent care to children have providers with the requisite competence to do so. Future work should focus on the generalizability of these competencies and curriculum to other settings and providers as well as examine the downstream effect on clinical practice and patient outcomes resulting from this training.

## Author Contributions

The manuscript was primarily created by authors CF and KS with review and input from the additional authors. CF: Pediatric Emergency Medicine Fellow Ann and Robert H. Lurie Children’s Hospital of Chicago; Massachusetts General Hospital for Children. KS: Attending, Pediatric Emergency Medicine and Hospitalist, Massachusetts General Hospital for Children. HP: Attending, Emergency Medicine, Massachusetts General Hospital; Global Health Fellow, Division of Global Health and Human Rights, Department of Emergency Medicine, Massachusetts General Hospital. KF: Attending, Pediatrics, North Shore Medical Center; Global Health Fellow, Division of Global Health and Human Rights, Department of Emergency Medicine, Massachusetts General Hospital. BN: Attending, Pediatrics, Massachusetts General Hospital. KO: Lecturer, Maseno University, and Sagam Community Hospital. TB: Chair of the Division of Global Health and Human Rights, Department of Emergency Medicine, Massachusetts General Hospital.

## Conflict of Interest Statement

The authors declare that the research was conducted in the absence of any commercial or financial relationships that could be construed as a potential conflict of interest.

## Abbreviations

AFEM, African Federation of Emergency Medicine; EM, emergency medicine; ETAT, emergency triage, assessment, and treatment; FEM, family and emergency medicine; IFEM, International Federation of Emergency Medicine; MGH, Massachusetts General Hospital; OSCE, observed structured clinical exam; PEM, pediatric emergency medicine.

## Appendix A

Kern et. al. model for curriculum development

Step 1. Problem Identification and General Needs Assessment
Step 2. Targeted Needs Assessment
Step 3. Goals and Objectives
Step 4. Educational Strategies
Step 5. Implementation
Step 6. Evaluation and Feedback
